# Aneurism of the Femoral Artery from Fracture of the Femur at Its Superior Part—Ligature of the External Iliac Artery—Recovery

**Published:** 1844-09

**Authors:** Daniel Brainard

**Affiliations:** Professor of Anatomy and Surgery in the Rush Medical College; Chicago, Ill.


*Aneurism of the Femoral Artery from fracture of the Femur at its superior part—Ligature of the external Iliac Artery—Recovery.* By Daniel Brainard, M. D., Professor of Anatomy and Surgery in the Rush Medical College.

March 1st, 1842, I was called to visit Dexter Graves, a highly respectable citizen of Chicago, Illinois, laboring under an ununited fracture of the femur. He gave the following history of the case: Twelve weeks previously, in the early part of December, 1841, while stepping from a carriage, he fell, in such a manner as to strike the upper part of the right thigh, at the inside, across the wheel. He was unable to rise, and a physician being called diagnosticated a "fracture of the bone." Provisional dressings were applied and the patient conveyed a distance of forty-five miles, to his own house in Chicago. Another apparatus was then applied by which extension and counter extension were kept up, and the limb kept in a straight position; this treatment was continued twelve weeks, when the machine was removed and the fracture found to be ununited. At this time I saw him.

The following were the appearances observed:—The right limb was two inches shorter than the left, the foot was everted, and considerable swelling existed about the hip and thigh. The foot could easily be brought down to a level with the other, but was immediately retracted on removing the extension. No crepitation could be perceived in any movement of the member. The shaft of the bone to near the trochanters could be felt, free from callus or fracture. The diagnosis was a fracture of the neck of the bone, but whether within or without the capsule there was no means of ascertaining with certainty. The general health of the patient had suffered much in consequence of his long confinement. He was pale, emaciated, affected with diarrhœa, loss of appetite, and profuse sweats. He had cough, expectoration streaked with blood; an examination of the chest revealed an extensive bronchial affection, but no signs of tubercularization.

Under these circumstances it was thought advisable to adopt some plan which would allow him to rise and take exercise, the restoration of his health being the most urgent indication. This was a thing of some difficulty, as the slightest movement was attended with excruciating pain. The immovable apparatus (starch bandage) was chosen and applied from the knee upward. Over a dry roller were placed several pieces of firm pasteboard, which externally and posteriorly extended as high as the crest of the ilium. These were covered with a roller immersed in paste of flour, several turns of which were passed around the pelvis. As soon as this was dried the patient was able to rise and walk on crutches, and in a short time was able to ride several miles. No change took place in the state of the limb except that at times it was painful and affected with spasmodic action of the muscles, when it was fully three inches shorter than its fellow. It may be remarked that the patient had felt pains in this limb of a rheumatic character previously to the fracture, they were however but slight and occasional. He remained in the same condition six months, wearing the starch bandage, which he could not do without, and with it I left him Oct. 20, 1842, to be absent at St. Louis during the winter. On my return, Feb. 17, 1843, (which was hastened by urgent applications on the part of the patient,) I found the upper part of the thigh occupied by a large tumour, the most projecting point of which was three inches below and a little anterior to the trochanter major. It was smooth, elastic, fluctuating, distinctly pulsating, giving the "*bruit de souffle*" on the application of the ear, subsiding in a marked degree on compression of the femoral artery, and becoming tense on removal of the pressure. Its inner margin was limited by the gracilis muscle, its outer at the most external part of the thigh; it extended upward to Poupart's ligament, and downward from that point twelve inches: the circumference of the thigh, embracing the largest part of the tumour, was twenty-five inches. It had been first perceived about twelve weeks previously, and rendered it necessary to remove the paste bandage and had increased regularly until it acquired its present size. If the characters I have described could leave any doubt of the aneurismal nature of this disease this had been entirely removed by the exploration of the physicians in attendance, who, supposing it to be an abscess, had made a puncture through which a pint of arterial blood had escaped before they could succeed in closing it. Compression of the femoral artery by means of a steel spring and graduated compress was then resorted to, but with no other sensible effect than the production of œdema of the limb from the obstruction of the venous and lymphatic circulation. The situation of the tumour, on the anterior and external side of the thigh, renders it probable that the anterior circumflex branch of the profound femoral artery was the one originally effected, but the course of the superficial femoral could not be traced, at the period when I first examined it, along the inside of the tumour.

Having observed the progress of the tumour for several days, in spite of the efforts made to check it, I determined on the ligature of the external iliac artery, and, the patient being in every respect in a favorable state, it was performed Feb. 24, 1843, in presence of several of the profession, and with the assistance of Drs. Sawyer and Davisson of this place, in the following manner:

The patient remaining upon his ordinary bed, with his head and shoulders slightly raised, an incision was made three inches in length, commencing an inch within the anterior superior spine of the ilium, and directed, parallel to Poupart's ligament, toward the pubis. The skin, subcutaneous tissue, and superficial fascia were thus divided, and the aponeurosis of the external oblique muscle exposed. The *arteria ad cutem* required a ligature. The aponeurosis was next divided upon a director, the internal oblique and transversalis muscles with the spermatic cord were pressed upward with the fingers, the handle of the scalpel was employed to rupture the fascia transversalis, and the peritoneum was then gently raised with the fingers until the forefinger of the left hand rested upon the artery about two inches above the femoral arch. The slight investment of the artery was penetrated by the nail, and the needle known as Dr. Pysick's, armed with a small silken ligature, passed under it. This was effected without difficulty, the point of the needle being carried between the artery and vein and pressed forward by gentle movements to and fro until it emerged on the outside of the artery. The point was then detached from the shaft and drawn out without embracing more than the artery itself, and without having raised or separated it from its surrounding tissues. The ligature was firmly tied in a double knot, one of its ends removed, the wound carefully sponged and its edges brought together by strips of adhesive plaster. Having had occasion during the winter, and previously to this operation, to put in practice, upon the dead subject, the different methods of placing a ligature upon this artery, I adopted the above mode of proceeding, as affording easy access to the vessel, with the least possible liability to wound the epigastric artery or the peritoneum, or to allow a protrusion of the viscera. Its application in this case fully justified the choice, and in no respect could I wish to have modified it.

During the operation the patient suffered very great pain, was pale, cold and depressed, and was allowed two glasses of wine. When it was finished the pain still continued, passing with rapidity from the wound to the limb or to the abdomen, and was so severe as to make the patient cry out and toss himself about in the utmost agony. It was relieved by the sulph. morph. gr. ¼, repeated three timcs. The immediate effects of arresting the circulation in the artery were not perceptible in the general system, unless the pain and agitation could be attributed to it—the pulse sixty and feeble. The tumour immediately became flaccid, its circumference being one inch less than before. Three hours after the operation there was a sensation of numbness in the member, and it was colder than the other; the sensibility gradually returned, and at the end of six hours was perfect; a state of exalted sensibility followed and was so great that the contact of a piece of flannel could not be endured. The natural temperature returned with the sensibility, commencing first above and extending to the foot, but there was not observed at any time an unnatural elevation of the temperature, or that activity of the capillary circulation which has been noticed in many cases.

During the first five days after the operation no material change occurred either in the member or in the system. The temperature and the sensibility of the former were natural. The pulse was 75, no febrile reaction, the patient urinated without difficulty, and on the fourth day had a stool from a lavement. His sleep was quiet and his appetite good. On the fifth day there was a slight discharge of pus from the wound. From the fifth to the fifteenth day he remained in the same state, being free from pain and all the functions regularly performed. From 15th to the 23d day there were occasional pains and cramps in the affected limb, which were relieved by frictions and hot applications, and a troublesome cough, for which anodynes were prescribed. On the 23d day the ligature came away. The incision, however, continued to discharge pus for several days afterwards, and was not perfectly healed before the 45th day from the operation.

The tumour after the tenth day did not diminish in size, but remained stationary; frictions and the roller were applied, which latter could only be used at first with moderate force; by degrees the pressure was increased and the tumour gradually subsided. A covering fitted to its surface and laced on the outside, attached to a band about the pelvis, and the sides of which were rendered stiff by the introduction of pieces of thin pasteboard, was at length substituted for the roller, and was found more effectual in hastening the disappearance of the tumefaction. At present, on the 1st of June, this had entirely been effected, there remained only an induration in its place, which extends over the whole anterior and superior femoral region, and confining the fragments and limiting their movements in such a manner, as to give the patient great command over the member. It can be moved freely in every direction, and is capable of sustaining a great part of the weight of the body, the patient being able to move about and attend to his business. Whether a bony union may still be expected, must depend in a great measure upon the seat of the fracture; if this is within the capsule it is not to be anticipated; if without, it may take place. That this latter is the case there are strong reasons for supposing; not, however, enabling us to adopt the opinion without reserve.

*Remarks.*—The appearance of aneurism as a consequence of fracture of the femur has not been often observed, and its occurrence is calculated therefore to direct attention to the time and manner of its production. In the present case there are three several ways in which this may be explained. By supposing the artery to have been injured by the violence which occasioned the fracture. Neither the manner of the fall nor the position of the limb after it, would justify us in adopting this explanation in the present instance. By the alternate elongation and contraction of the member; this, carried to the extent of three inches, and repeated frequently during a period of several months, might be supposed capable of impairing the integrity of the coats of the artery, but only after the lapse of a certain period of time, whereas in this case we have indications of its earlier existence. These indications were the unusual tenderness, the persisting tumefaction, the absence of bony union, although the value of this latter circumstance was diminished by the unsuitable apparatus employed, the improper manner of its application, and the doubt as to the precise point of the fracture. Still the position of the tumour, rendering it almost certain that its origin was from the profunda or its branches, coupled with the former cousiderations, will justify us in attributing it to the transportation of the patient, immediately after the fracture, so great a distance.

The laceration of the tissues, necessarily produced by the movements of a carriage, over a very rough road, a distance of 45 miles, must be very great, about the fractured ends, and to this we may with great probability ascribe the production of the aneurism in this instance; and from it we may derive a practical precept of caution in advising or permitting such a transportation in any similar case.

Chicago, Ill., June 15, 1843.

*American Journal, Oct.* 1843.

